# Gender influence on mortality and outcome of severe sepsis patients in intensive care unit

**DOI:** 10.1186/2197-425X-3-S1-A965

**Published:** 2015-10-01

**Authors:** C Wullur, I Wiraatmaja, E Oktaliansah, T Maskoen

**Affiliations:** Universitas Padjajaran, Department of Anaesthesia and Intensive Care, Bandung, Indonesia

## Introduction

The influence of gender on outcome of patients with severe sepsis is unclear. Different concentrations of sex steroid hormone has been postulated to determine the effect of gender on outcome.

## Objectives

To investigate the impact of gender on mortality and length of intensive care unit (ICU) stay of severe sepsis patients treated in ICU.

## Methods

This is a prospective cohort study involving severe sepsis patients admitted to ICU Hasan Sadikin General Hospital Bandung Indonesia between period of August 2013 - January 2014. Data of type of admission, initial Sequential Organ Failure Assessment (SOFA) score, mortality and length of stay (LOS) in ICU were collected. Severity of illness was measured using Acute Physiology and Chronic Health Evaluation (APACHE) II.

## Results

Of the 150 patients included in the study, 66.7% were male. There was no difference in age distribution between male and female patients. Most of the patients admitted was surgical patients (84%). ICU 28 days mortality was higher in male compared to female patients (61.3% vs. 52.6%), the difference is not statistically significant (p = 0.547). However, when comparing between surgical and medical admission, surgical male patients are at significantly higher risk of mortality compared to female (relative risk [RR]: 2.7, 95% confidence interval [CI]: 1.112 to 6.598) (p = 0.033). Slightly more men had high APACHE II score (≥ 17) compared to women (90% vs. 77.8%) (p = 0.582). Compared to women, men also had longer LOS in ICU (12.7 ± 6 vs. 10 ± 6.2 days) (p = 0.19) and higher SOFA score (6.7 ± 2.3 vs 6.0 ± 2.86) (p = 0.371).

## Conclusions

Gender contributes to poor outcome in postoperative ICU patients. Surgical male patients had higher risk of mortality from severe sepsis compared to female (relative risk [RR]: 2.7) (p = 0.033). Male patients also had higher APACHE II score (p = 0.582), longer LOS in ICU (p = 0.19) and higher SOFA score (p = 0.371) compared to female patients.
